# Antimicrobial Drugs and Community-acquired Methicillin-Resistant *Staphylococcus aureus,* United Kingdom

**DOI:** 10.3201/eid1307.061561

**Published:** 2007-07

**Authors:** Verena Schneider-Lindner, J. A. Delaney, Sandra Dial, Andre Dascal, Samy Suissa

**Affiliations:** * McGill University Health Center, Montreal, Quebec, Canada; † Royal Victoria Hospital, Montreal, Quebec, Canada; ‡ Sir Mortimer B. Davis–Jewish General Hospital, Montreal, Quebec, Canada

**Keywords:** Methicillin-resistant Staphylococcus aureus, general practice research database, antimicrobial agents, epidemiology, case-control study, pharmacoepidemiology, risk factors, infectious disease, research

## Abstract

We report results of a case–control study of the association between receipt of antimicrobial agents and diagnosis of community-acquired methicillin-resistant *Staphylococcus aureus* (MRSA) in the United Kingdom. Eligible adults, selected from the General Practice Research Database, had no previous diagnosis of MRSA, no hospitalization in the past 2 years, and >2 years of follow-up recorded in the database. For 2000–2004, we identified 1,981 MRSA case-patients and 19,779 matched control-patients. The odds ratios (ORs) and 95% confidence intervals (CIs) of MRSA diagnosis for patients who were prescribed 1, 2–3, or >4 antimicrobial drugs were 1.57 (CI 1.36–1.80), 2.46 (CI 2.15–2.83), and 6.24 (CI 5.43–7.17), respectively. Risk for community-acquired MRSA increased with number of antimicrobial drug prescriptions, appeared to vary according to antimicrobial drug classes prescribed the previous year, and was highest for quinolones (OR 3.37, CI 2.80–4.09) and macrolides (OR 2.50, CI 2.14–2.91).

## Abstract 2

Antimicrobial drugs are associated with an increased risk for community-acquired MRSA infections.

Methicillin-resistant *Staphylococcus aureus* (MRSA) was detected in the United Kingdom in 1961, only months after methicillin introduction (*1*–*3*). Since then, MRSA has become a common cause of nosocomial infections worldwide (*1*–*3*). In 1993, MRSA infections emerging in the community were reported (*1*). MRSA infections acquired in the community differ from those acquired in the hospital with respect to their epidemiology and the characteristics of the causative MRSA strains (*1*,*2*,*4*–*6*).

The prevalence of colonization with MRSA has been established in various community populations (*7*–*9*). However, patient characteristics and risk factors for clinically significant MRSA infections acquired in the community have so far been described for specific outbreaks (*10*,*11*) or case series without an adequate population-based control group (*12*,*13*).

Nosocomial MRSA is associated with antimicrobial drugs and specific antimicrobial drug classes (*14*–*16*). The role of antimicrobial drugs in community-acquired MRSA is less clear. A recent study of 34 case-patients with community-acquired MRSA in Alaska showed that case-patients were more likely than control-patients to have received antimicrobial agents in the year before the outbreak (*10*). Whether risk for community-acquired MRSA differs according to exposure to agents from different antimicrobial drug classes is not clear. We therefore sought to describe the association between exposure to antimicrobial drugs and a subsequent diagnosis of MRSA, including exposure to individual antimicrobial drug classes.

## Methods

We conducted our retrospective case–control study by using the General Practice Research Database (GPRD). This primary care database contains the diagnostic, laboratory test, and prescribing records of ≈3.2 million patients from >400 general practices in the United Kingdom. The GPRD is used extensively for research on drugs (*17*,*18*) and has also been used for research on infectious diseases (*19*,*20*).

### Case-Patients and Control-Patients

Eligible participants were >18 years of age, had no previous diagnosis of MRSA, no hospitalization in the past 2 years, and >2 years of follow-up recorded in the GPRD. We excluded persons who had been recently hospitalized to ensure that we studied patients with community-acquired rather than community-onset MRSA.

We identified as case-patients all persons with a first clinical diagnosis of MRSA from January 1, 2000, through December 31, 2004. To include all possible codes that a general practitioner could use to diagnose MRSA in the GPRD, we considered the following Read Clinical Classification codes (now National Health Service Clinical Terms) to represent a diagnosis of MRSA: 4JP..00 (methicillin-resistant *Staphylococcus aureus* positive), SP25800 (MRSA infection of postoperative wound), and ZV02A00 ([V]MRSA-multiple resistant *Staphylococcus aureus* infection carrier). Because the most frequently entered code (4JP..00) does not explicitly differentiate between infection and colonization, we were unable to determine from the codes whether most patients in this study were infected or colonized. The date of the MRSA diagnosis was used as the index date for each case. Microbiologic test results were not used to identify case-patients with MRSA because results for such testing were not systematically available in the GPRD.

For each case-patient, we randomly selected 10 control-patients also from the GPRD, matched by general practice and age (±2 years). To control for calendar time, we assigned all control-patients their corresponding case-patient’s index date. Control-patients had to fulfill the same exclusion criteria as case-patients.

### Exposure

For patients in each group, we determined exposure to antimicrobial drugs 30–365 days before the index date. To avoid the possibility of protopathic bias, we excluded antimicrobial drug prescriptions made during the 29 days prior to the index date. We classified the number of antimicrobial drugs prescribed for each patient during this period into 4 categories: 0 (unexposed), 1, 2–3, and >4 prescriptions. For the same period, we defined 7 mutually exclusive categories for classes of antimicrobial drugs according to their British National Formulary (BNF) code (*21*): penicillins (5.1.1), cephalosporins (5.1.2), tetracyclines (5.1.3), macrolides (5.1.5), sulfonamides (5.1.8), quinolones (5.1.12), and an additional category of all other antimicrobial drugs. The “other antimicrobial drugs” category represented BNF antimicrobial drug categories that are infrequently prescribed (such as clindamycin) or for which the antimicrobial drug is part of a combination prescription listed in a nonantimicrobial drug BNF category.

### Covariates

We adjusted for age, sex, smoking, and obesity (body mass index >30) as relevant demographic and life style factors possibly associated with exposure to antimicrobial drugs and MRSA infections. We further controlled for a series of known risk factors and concurrent conditions diagnosed during the 2 years before the index date: heart disease (myocardial infarction, congestive heart failure), stroke, peripheral vascular disease, chronic obstructive pulmonary disease, liver disease, skin diseases (intertrigo, eczema, psoriasis), renal failure, cancer, autoimmune diseases (lupus, rheumatoid arthritis), previous infection with *Clostridium difficile*, and previous infection with *S. aureus* (susceptible to methicillin). These conditions were defined according to diagnostic codes. We defined diabetes according to prescribed insulin or a clinical diagnosis in the 2 years before the index date. Finally, because of its immunosuppressive effect, we considered oral prednisone (defined according to prescriptions) prescribed during the 1 year before the index date to be a potentially confounding drug.

### Data Analysis

For each year of the study period, we calculated the incidence rate of first MRSA diagnosis. The numerator consisted of the number of eligible case-patients each year; the denominator was all members of the GPRD population who were registered with general practices that met GPRD quality control standards and who were >18 years of age during that year.

We used conditional logistic regression to estimate the odds ratio (OR) of the association between antimicrobial drug prescriptions and a subsequent diagnosis of MRSA (*22*). For a rare outcome like MRSA, the OR is an approximation of the rate ratio. To obtain adjusted ORs, we repeated the analyses with covariates included in the regression model. In a separate analysis for the number of antimicrobial prescriptions, we included variables that represented the 4 categories we defined for the number of prescriptions. For our analysis of association according to different class of antimicrobial drug, we used a separate statistical model that contained variables for all 7 categories that represented the different classes of antimicrobial drugs.

In a sensitivity analysis of the 3 Read Clinical Classification codes of our case definition, we determined the association between antimicrobial drugs and MRSA diagnosis in 3 different models. In each model, we included only the subset of case-patients who had the appropriate code and their corresponding control-patients. We also included the covariates in each of the 3 models to obtain adjusted ORs.

We used SAS version 9.1.3 (SAS Institute, Cary, NC, USA) for all analyses. We obtained approval from the Scientific and Ethical Advisory Group of the GPRD and the McGill University Health Center Research Ethics Board at the Chest Hospital.

## Results

A total of 3,408 patients had a first diagnosis of MRSA in the GPRD during the study period. After exclusion of patients who did not fit the other criteria, 1,981 (58.1%) remained eligible for our study, for which we identified 19,779 matching control-patients. The MRSA diagnosis was recorded as medical code 4JP..00 for 85.5%, code SP25800 for 8.8%, and code ZV02A00 for 5.7% of case-patients.

During the study period, the annual number of case-patients with MRSA in the GPRD-based population rose from 332 to 484 (Figure). Average incidence of MRSA infections during the study period was 15.2 cases per 100,000 persons per year. The median age of case-patients during the study period was 74 years (interquartile range 59–83 years).

**Figure:**
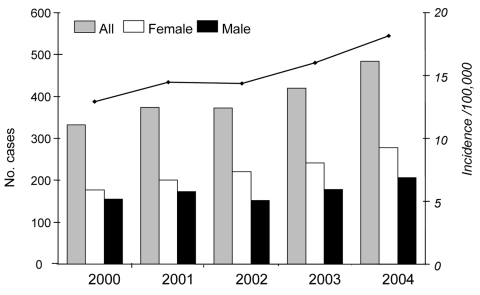
Annual number of all study participants with methicillin-resistant *Staphylococcus aureus* (MRSA) recorded for the first time in the General Practice Research Database (GPRD) and no hospitalization in the past 24 months (vertical bars). The annual incidence rate of MRSA per 100,000 adults in the GPRD is indicated by the line above the bars. Data from the GPRD, United Kingdom, 2000–2004.

Overall, concurrent conditions were diagnosed more frequently for case-patients than for control-patients, and oral prednisone was prescribed more often for case-patients (Table 1). Smoking status did not differ significantly (adjusted OR 1.06, 95% CI 0.95–1.17), but more case-patients than control-patients were recorded as obese (OR 1.27, 95% CI 1.10–1.45).

**Table 1:** Characteristics of patients with (case-patients) and without (control-patients) a diagnosis of community-acquired methicillin-resistant Staphylococcus aureus*

Characteristic	Case-patients (N = 1,981), no. (%)	Control-patients (N = 19,779), no. (%)	Crude OR	Adjusted OR†	95% CI
Age, y (SD)	69.4 (18.4)	69.2 (18.5)	1.07	1.08	1.07–1.12‡
Male	864 (43.6)	8,429 (42.6)	1.05	1.08	0.98–1.20

Concurrent conditions					
Diabetes	247 (12.5)	1,195 (6.0)	2.27	1.70	1.44–2.00‡
MI and heart failure	117 (5.9)	563 (2.8)	2.22	1.42	1.13–1.80‡
Stroke	95 (4.8)	212 (1.1)	4.64	4.09	3.13–5.35‡
Peripheral vascular disease	49 2.5)	92 (0.5)	5.49	3.65	2.49–5.35‡
COPD	117 (5.9)	465 (2.4)	2.68	1.45	1.13–1.85‡
Skin diseases (intertrigo, eczema, psoriasis)	196 (9.9)	1,332 (6.7)	1.53	1.19	1.01–1.41‡
Renal failure	65 (3.3)	188 (1.0)	3.64	2.53	1.88–3.50‡
Cancer	42 (2.1)	183 (0.9)	2.39	2.01	1.39–2.91‡
Liver disease	6 (0.3)	8 (0.04)	7.50	6.69	2.17–20.66‡
Autoimmune diseases	17 (0.9)	93 (0.5)	1.84	1.27	0.72–2.25
*Clostridium difficile*	9 (0.5)	15 (0.1)	6.20	5.33	2.13–13.35‡
*S. aureus* susceptible to methicillin	8 (0.4)	14 (0.1)	5.71	2.98	1.13–7.83‡
Oral prednisone use	216 (10.9)	1,163 (5.9)	1.99	1.11	0.93–1.33

Lifestyle factors					
Smoking	718 (36.2)	6,365 32.2)	1.21	1.06	0.95–1.17
Obesity	323 (16.3)	2,253 (11.4)	1.54	1.27	1.10–1.47‡

*Data from the General Practice Research Database, United Kingdom, 2000–2004. OR, odds ratio; CI, confidence interval; MI, myocardial infarction; COPD, chronic obstructive pulmonary disease. †Adjusted for all other variables in the table and exposure to antimicrobial drugs in the 30–365 days period before the index date. ‡p<0.05.

Exposure to any antimicrobial drug in the 30–365 days before index date, regardless of drug class and number of prescriptions, was associated with ≈3-fold risk of a MRSA diagnosis when compared with lack of such exposure (OR 2.61, 95% CI 2.36–2.89, Table 2). Among case-patients, 38.9% had not received any antimicrobial drug prescription during this period.

**Table 2:** Risk for infection with community-acquired methicillin-resistant *Staphylococcus aureus* (MRSA)*

No. prescriptions of antimicrobial drugs	Case-patients (N = 1,981), no. (%)	Control-patients (N = 19,779), no. (%)	Crude OR	Adjusted OR†	95% CI
0 (reference)	770 (38.9)	12,821 (64.8)	1	1	
>1	1,211 (61.1)	6,958 (35.2)	2.98	2.61	2.36–2.89
1	328 (16.6)	3,306 (16.7)	1.69	1.57	1.36–1.80
2 or 3	373 (18.8)	2,389 (12.1)	2.73	2.46	2.15–2.83
>4	510 (25.7)	1,263 (6.4)	7.27	6.24	5.43–7.17

*For persons prescribed <1 antimicrobial drug in the 30–365 days before their index date, relative to risk for MRSA infection in persons with no prescriptions during the same period. Data from General Practice Research Database, United Kingdom, 2000–2004. OR, odds ratio; CI, confidence interval. †Adjusted for all variables in Table 1.

The association of antimicrobial drugs and MRSA was stronger for persons who had received more prescriptions of any class of antimicrobial drug (Table 2). A substantial proportion of case-patients (25.7%) had received >4 prescriptions. This larger number of prescriptions was associated with a ≈6-fold increase in the risk for MRSA compared with the risk for persons who had not received any antimicrobial drug prescriptions (OR 6.24, 95% CI 5.43–7.17).

Individual classes of antimicrobial drugs were differentially associated with a diagnosis of MRSA (Table 3). The association was strongest for macrolides and quinolones; adjusted ORs were 2.50 (95% CI 2.14–2.91) and 3.37 (95% CI 2.80–4.09), respectively. This association means that risk for an MRSA diagnosis triples for persons who received >1 prescription of a quinolone, regardless of how many other antimicrobial drugs were prescribed for this patient during the 30–365 days before the diagnosis. We could not establish an association between tetracycline prescriptions and MRSA in our study.

**Table 3:** Risk for infection with community-acquired methicillin-resistant *Staphylococcus aureus* (MRSA)*

Class of antimicrobial drug	Case-patients (N = 1,981), no. (%)	Control-patients (N = 19,779), no. (%)	Crude OR	Adjusted OR†	95% CI
No prescription (reference group)	770 (38.9)	12,821 (64.8)	1	1	
Cephalosporins	27 13.6 ()	994 (5.0)	2.01	1.85	1.57–2.19
Macrolides	32 (16.2)	1,04 (5.2)	2.70	2.50	2.14–2.91
Penicillins	540 (27.3)	3,104 (15.7)	1.67	1.56	1.39–1.76
Other antimicrobial drugs	204 (10.3)	1,615 (8.1)	2.02	1.90	1.61–2.24
Sulfonamides	260 (13.1)	1,114 (5.8)	1.77	1.74	1.48–2.04
Tetracyclines	77 (3.9)	461 (2.3)	1.14	1.09	0.83–1.43
Quinolones	218 (11.0)	434 (2.2)	3.81	3.37	2.80–4.09

*For persons prescribed different classes of antimicrobial drugs in the 30–365 days before their index date, relative to risk for MRSA infection in persons with no prescriptions during the same period. Data from General Practice Research Database, United Kingdom, 2000–2004. OR, odds ratio; CI, confidence interval. †Adjusted for prescriptions from other antimicrobial drug classes in the 30–365 days before the index date and all variables in Table 1.

The results of our sensitivity analysis for diagnostic codes are presented in Table 4. Despite some variation in the estimated OR between the different methods of coding the MRSA diagnosis, we found an association with antimicrobial drugs, regardless of the code used to record the MRSA diagnosis.

**Table 4:** Sensitivity analysis for the clinical code used to define methicillin-resistant *Staphylococcus aureus* (MRSA)*

Clinical code†	Case-patients		Control-patients		Crude OR	Adjusted OR‡	95% CI
	N (%)	% Exposed	N	% Exposed			
Any code	1,981 (100)	61.1	19,779	35.2	2.98	2.61	2.36–2.89
4JP..00	1,735 (85.5)	62.2	17,327	35.7	3.05	2.66	2.39–2.97
SP25800	157 (8.8)	60.5	1,570	32.7	3.34	2.99	2.06–4.33
ZV02A00	113 (5.7)	47.8	1,122	30.5	2.09	1.98	1.30–3.01

*Risk for MRSA diagnosed in the community for persons with any number of antimicrobial drug prescriptions (exposed) in the 30–365 days before their index date relative to risk for MRSA for persons with no prescriptions during the same period. Data from 4 different analyses of a matched case-control study of patients listed in the General Practice Research Database, UK, 2000–2004. OR, odds ratio; CI, confidence interval. †4JP..00, MRSA positive; SP25800, MRSA infection of postoperative wound; ZV02A00, [V]MRSA multiple-resistant *Staphylococcus aureus* infection carrier. ‡Adjusted for all covariates in Table 1.

## Discussion

This study provides evidence of an association between previous antimicrobial drug prescriptions and a diagnosis of MRSA in the community. This association appears to be dose-dependent and to vary according to antimicrobial class; it is particularly strong for previous exposure to fluoroquinolones and macrolides. A substantial proportion of case-patients, however, were not prescribed antimicrobial drugs in the year before MRSA diagnosis. Persons who had concurrent conditions and persons who were obese were at higher risk for MRSA.

The clear dose-response relationship and the differential associations across antimicrobial drug classes support an association of antimicrobial drugs and MRSA in the community. This association is consistent with nosocomial MRSA, in which the use of specific antimicrobial drugs is linked to antimicrobial resistance. Previous studies have found fluoroquinolones, macrolides, and cephalosporins to be associated with nosocomial MRSA (*14*–*16*,*23*). Moreover, a dose-dependent association exists between exposure to antimicrobial drugs and nosocomial MRSA on the patient level and on the hospital level (*16*).

In a US surveillance study, incidence of community-acquired MRSA was 25.7 case-patients per 100,000 in Atlanta and 18.0 case-patients per 100,000 in Baltimore, findings that are highly consistent with ours (*12*). In that study, a considerable proportion of cases occurred in persons >65 years of age. In our study, compared with previous outbreak reports (*10*,*11*), case-patients were older and had more concurrent conditions. This finding is likely because we did not include any cases in children and, in contrast to reports of outbreaks, our cases are sporadic and thus less prone to be reported.

Similarly, specific occupation-related risk factors (e.g., abrasions, crowded housing) are likely to be more prevalent in a study based on military beneficiaries compared with a study based on the general population. This could explain the higher incidence of MRSA infections found in such a study (*13*) than in ours.

Our study population was a representative sample of the UK general population (*17*). The use of the GPRD therefore enables the examination of a large number of MRSA infections diagnosed by general practitioners in the community. However, the GPRD has some limitations for the study of an infectious disease. We lacked information on severity and site of MRSA infection and on patient lifestyle characteristics (e.g., incarceration, intravenous drug use). We also lacked information on molecular characteristics of the MRSA strains and thus cannot exclude the possibility that MRSA was diagnosed in cases that did not result from between-patient spread within the community, but rather from secondary exposure to the hospital environment through family members, visitation, or employment. However, the importance of the lack of microbiologic information on the causative MRSA strains may be questioned because it no longer enables a distinction to be made between community and nosocomial MRSA strains (*24*).

The clinical codes that we used for our case definition are likely to be specific, but they may lack sensitivity; some MRSA infections that would have met the Centers for Disease Control and Prevention’s case definition of infection may not be captured with the clinical codes. This lack of sensitivity may affect risk factors for MRSA that we observed in this study as well as the strength of the association between antimicrobial drugs and later MRSA diagnosis.

To our knowledge, MRSA infections in the GPRD have not been previously studied, although infectious diseases in this database have been, such as acute respiratory infections (*19*), urinary tract infections (*20*), *C. difficile* infections (*25*), pneumonia (*26*), and sexually transmitted infections (*27*). Disease codes for clinically relevant outcomes in the GPRD have been validated (*17*); however, MRSA has not been included in such studies.

In previous UK community studies, ≈0.8% of elderly participants were colonized with MRSA (*9*,*28*). In a meta-analysis, the pooled MRSA colonization rate was 1.3% (*8*). In persons without prior health care contacts, the rate was 0.2% (*8*). We report an incidence rate of MRSA diagnosis in the community that is too low to be consistent with these prevalence figures, probably because general practitioners are unlikely to screen asymptomatic patients for colonization with MRSA.

The results of our sensitivity analysis of the 3 diagnostic codes we used to define these MRSA cases suggest that antimicrobial drugs promote both MRSA infection and colonization. The association between antimicrobial drugs and MRSA diagnosis was weaker in case-patients with a diagnosis of carrier status and stronger in those with a postoperative wound infection. The association observed in the patients who had their diagnosis recorded with the most frequently used code (4JP..00) appeared to be more similar to those coded as postoperative wound infections (SP25800) than to those coded as carriers (ZV02A00). This finding supports the hypothesis that active infections are more likely than colonization to be recorded in a general practice database. Therefore, separating risk factors for acquiring MRSA from risk factors for increased severity of infection with MRSA using the approach of this study may be difficult.

We did not consider antimicrobial drugs that were prescribed in the 30 days before MRSA diagnosis. With this exposure definition, we prevent mistaking prescriptions issued for treatment of the infection as causes of the infection (protopathic bias [*29*]). This distinction is relevant for MRSA, for which the diagnosis is likely delayed due to outstanding microbiologic test results or likely made after failure of empirical treatment with antimicrobial drugs. Therefore, we may have wrongly classified as unexposed some persons whose exposure to antimicrobial drugs in fact preceded the MRSA infection. Any bias resulting from this exposure definition will be toward the null hypothesis and thus will weaken the effect of antimicrobial drugs as promoters of MRSA infections.

To minimize the chances of overlooking any important confounders, we adjusted our analyses for age, sex, lifestyle factors, and a broad range of concurrent conditions. That antimicrobial drug prescriptions are a marker for an important unknown confounder is remotely possible. However, to substantially bias our results, such a confounder would need to be strongly related to both the prescription of antimicrobial drugs and the diagnosis of MRSA but unrelated to our study covariates.

Further support for our results comes from the ecologic association between fluoroquinolone use and nosocomial MRSA found in an intervention study (*30*) and a quasi-experimental study (*31*). Similar to nosocomial MRSA (*16*,*23*), the use of specific antimicrobial drugs in the community may cause selection pressures that favor the acquisition of resistance in *S. aureus* on the community level.

In conclusion, the role of antimicrobial drugs in MRSA diagnosed in the community appears to be similar to their role in nosocomial MRSA. Therefore, appropriate use of antimicrobial drugs, in addition to traditional infection control measures, may be a strategy to not only control nosocomial MRSA (*32*), but also to limit the incidence of community-diagnosed MRSA infections.
